# Professional values at the beginning of medical school: a quasi-experimental study

**DOI:** 10.1186/s12909-024-05186-8

**Published:** 2024-03-08

**Authors:** Sandra Vilagra, Marlon Vilagra, Renata Giaxa, Alice Miguel, Lahis W. Vilagra, Mariana Kehl, Milton A. Martins, Patricia Tempski

**Affiliations:** 1 Faculdade de Medicina de Vassouras, Vassouras, Brazil; 2 Centro de Desenvolvimento de Educação Médica, Faculdade de Medicina da Universidade de São Paulo, São Paulo, Brazil; 3 Universidade Federal de São Carlos, São Carlos, Brazil

**Keywords:** Critical incident, COVID-19, Professionalism, Narratives, Professional identity, Self-reflection

## Abstract

**Background:**

Teaching professionalism in medical schools is central to medical education and society. We evaluated how medical students view the values of the medical profession on their first day of medical school and the influence of a conference about the competences of this profession on these students’ levels of reflection.

**Methods:**

We studied two groups of medical students who wrote narratives about the values of the medical profession and the influence of the COVID-19 pandemic on these values. The first group wrote the narratives after a conference about the competences of the medical profession (intervention group), and the second group wrote the same narratives after a biochemistry conference (control group). We also compared the levels of reflection of these two groups of students.

**Results:**

Among the 175 medical students entering in the 2022 academic year, 159 agreed to participate in the study (response rate = 90.8%). There were more references to positive than negative models of doctor‒patient relationships experienced by the students (58.5% and 41.5% of responses, respectively). The intervention group referred to a more significant number of values than the control group did. The most cited values were empathy, humility, and ethics; the main competences were technical competence, communication/active listening, and resilience. The students’ perspectives of the values of their future profession were strongly and positively influenced by the pandemic experience. The students realized the need for constant updating, basing medical practice on scientific evidence, and employing skills/attitudes such as resilience, flexibility, and collaboration for teamwork. Analysis of the levels of reflection in the narratives showed a predominance of reflections with a higher level in the intervention group and of those with a lower level in the control group.

**Conclusions:**

Our study showed that medical students, upon entering medical school, already have a view of medical professionalism, although they still need to present a deeper level of self-reflection. A single, planned intervention in medical professionalism can promote self-reflection. The vision of medical professional identity was strongly influenced by the COVID-19 pandemic, positively impacting the formation of a professional identity among the students who decided to enter medical school.

## Background

Professionalism is essential to society’s trust in the medical profession and is central to the doctor‒patient relationship [1–3]. Professionalism includes placing the interests of patients above those of physicians and acting according to high standards of competence and integrity [3]. In line with the American College of Physicians and other internal medicine societies, professionalism includes many professional responsibilities, such as commitment to professional competence, honesty with patients, patient confidentiality, maintaining appropriate relations with patients, improving quality and access to care, just distribution of finite resources, scientific knowledge, managing conflicts of interest and professional responsibilities [3, 4].

The development of medical professionalism in undergraduate medical education is a complex enterprise that involves substantial compromise with society in general and with a person individually [5, 6]. Developing and teaching professionalism in medical schools has been considered central to medical education and has been the subject of many previous studies [7, 8]. However, to better teach professional values, it is imperative to know what students think and know about the values of the medical profession when starting medical school.

The years of the COVID-19 pandemic had a significant influence on how society views the medical profession and its values, and medical students who started medical school in 2022 or 2023 were strongly influenced by the changes imposed on society and schools, at least in countries that had a substantial impact of COVID-19 [9, 10].

In the present study, we evaluated how medical students view the values of the medical profession on their first day of medical school. We studied two groups of medical students who wrote narratives about the values of the medical profession and the influence of the COVID-19 pandemic on their perceptions of these values on the first day of medical school. The first group wrote the narratives after a conference about the competences of the medical profession, and the second group wrote the same narratives after a biochemistry conference. Our goal was to evaluate the values of professionalism that medical students consider important before starting medical school and the impact of one intervention on medical professionalism on these responses.

## Methods

We performed a quasi-experimental study involving 175 first-year medical students in the School of Medicine of the University of Sao Paulo (USP), Brazil.

The research ethics committee of the School of Medicine of the University of Sao Paulo approved this study (Protocol 56043522.9.0000.0068). Participation was voluntary, without compensation or incentives. We guaranteed confidentiality and anonymity. Data collection was performed in March 2022, on the first day of the medical program, after the participating students completed a written consent form. The study was performed in the classroom using notebooks and/or cell phones to access the electronic forms during regular academic activity.

### Study design

We divided the students into two groups. The control group (CG) attended a biochemistry class before study participation, and the intervention group (IG) attended a lecture about “The competences of a physician in the 21st century” and then was asked to participate in the study.

At the conference, the competency framework of the Royal College of Physicians and Surgeons of Canada (CanMeds) was presented [11]. This framework includes medical expert, communicator, collaborator, leader, health advocate, scholar, and professional general competences. In this conference, general competences of the 21st century, including critical thinking, collaboration, communication, creativity, connectivity, and respect for diversity, were also discussed.

Both groups had two hours to complete four narratives:

1. “Tell us something that happened to you in your life that influenced your view of the medical profession.”
2. “What are, in your opinion, the main medical profession values?”
3. “How do you imagine yourself working as a medical doctor ten years after graduation?”
4. “How did the COVID-19 pandemic influence your view of the medical profession and your future practice?”

At the end of the study, the control group attended the medical education class, and the intervention group attended the biochemistry class.

### Critical incident reports and self-reflection

The first narrative (“Tell us something that happened to you in your life that influenced your view of the medical profession”) was a critical incident report. Critical incident reports are short personal narratives about the experiences of medical students, residents, and other learners that can be used to address values and attitudes and discuss professionalism [12, 13]. These narratives focus on the meaning of these experiences for developing professional identity and personal growth [14]. The use of critical incidents is a reflective method that can potentially enhance critical thinking [12].

We used critical incident reports and the other narratives to analyze the impacts of life events and their meaning, the role of the COVID-19 pandemic in the students’ decisions to be physicians, and their expectations and vision of the values of the medical profession and health care.

### Data analysis

We used the COREQ model to assess the quality of the research process and data analysis [15]. The qualitative analysis was based on the students’ narratives. These narratives were prepared for analysis and categorized according to traditional content analysis methods [16–18]. Three independent researchers started with a free reading of the transcribed text without trying to categorize it. During the second reading, the researchers categorized themes and derived issues separately. Finally, each researcher’s products were paired by similarities in meaning and were discussed with the research group. The results were divided into analytical categories, items, and examples. The same group performed the reflection analysis, which started with individual classification, followed by group discussion.

In our study, we chose to analyze the medical students’ reflections using the framework of Nieme [19], which includes four levels of reflection: (1) committed reflection, in which one discusses meanings and learnings from experience and how they have affected his or her professional identity or practice and provides some evidence; (2) emotional exploration, which is characterized by emotional expressions and self-awareness, showing some signs of reflection; (3) objective reporting, in which one presents facts that he or she acted as a participant or observer, without or with few expressions of their reactions and the elaboration of the experiences; and (4) scant and avoidant reporting, which consists of short or superficial reporting.

We used the chi-square test to compare the levels of self-reflection between the two groups (questions 1, 3 and 4) and answers to question 2. The significance level was established at 0.05.

## Results

Among the 175 medical students entering in the 2022 academic year, 159 agreed to participate in the study (response rate = 90.8%). Sixteen students were not included because they were absent at both collection moments. No student who was present refused to participate in the survey. The age of the participants ranged from 17 to 39 years (average = 20.3 ± 3.6 years); 90 were males (56.6%), and 69 were females. The percentage of male students was similar in both groups, with 57.3% and 55.1% in the intervention group and the control group, respectively.

The analysis of the answers to the question “Tell us something that happened to you in your life that influenced your view of the medical profession” generated three categories: “Process of illness and care”, “Role models”, and “Previous correlated experiences” (Table 1). In the category of “Process of illness and care”, emphasis was placed on the positive and negative models of the doctor‒patient relationship that were experienced, with the positive models being sources of inspiration and the negative models being catalysts for entering the profession and changing the current scenario. We observed that positive models were more frequent than negative models (58.5% and 41.5% of responses, respectively). Medical performance in the COVID-19 pandemic was included as a specific item, given the frequency with which the participants highlighted it. Interprofessional care and the idea that doctors do not work in isolation were highlighted, as shown in the following statement:

*“The pandemic has further highlighted medical dedication and the importance of interprofessional service collaboration…” (S44, female, 20 years old, IG)*

**Table 1:** Qualitative analysis of responses to the question “Tell us something that happened to you in your life that influenced your view of the medical profession.”

Category	Items	Sample Speeches
Process of illness and care	Personal experience of illness	*“I suffered an accident at a birthday party. During the confusion, a doctor was proactive and soon took the lead, managing to stop the injury quickly and driving me to the hospital, constantly reassuring me and giving me confidence”. (S4, female,18 years old, IG)*
	Family members’ illness	*“Being with my mother, I could see the impact each doctor had on her life, both in terms of her health and the way she viewed her illness”. (S124, female,18 years old, CG)*
	Medical care	*“When I was 14, my grandfather spent many months in a hospital. Seeing the dedication of the doctors to him and all the knowledge they brought to accomplish their profession, I became interested in the medical field…” (S87, female, 21 years old, IG)*
	Experience in the pandemic	*“When my father had to be hospitalized due to covid, to the point of being intubated, I saw how solicitous and sensitive the new generation of doctors was with me who received information about what was happening to him…” (S133, male, 24 years old, CG)*
Role models		
	Professional	*“During the guided visit to the Faculty of Medicine in 2018, I realized how serious and responsible the students and professors are, resulting from what the profession demands. In addition, the cultural environment of this faculty showed me, in practice, that the daily life of a doctor is made up of a lot of study.” (S16, female, 21 years old, IG)*
	Management	*“Participation in discussion groups on technology, leadership, management, and entrepreneurship in healthcare. It allowed me to learn about the medical market and its trends”. (S21, male, 19 years old, IG)*
	Teachers	*“I ended up being captivated by the physiology lessons that a physical education teacher taught at school. From then on, I began to seriously consider pursuing a medical career…” (S88, male,19 years old, CG)*
	Literature	*“A book I was reading at the time, whose main character was a doctor who talked about his motivations, filled my eyes with the possibility of the humanitarian action of medicine”. (S79, male, 18 years old, IG)*
	Cinema	*“In particular, I cite two films that served as significant and influential events: Path Adams (especially regarding humanization in the profession) and St. Giuseppe Moscati (who treated everyone with the differential of love and genuine attention).” (S63, female, 19 years old, IG)*
Previous correlated experiences	Visiting colleges and hospitals	*“I did the workshop organized by the scientific department of the college in which I would have an anatomy class with authentic pieces […] I was amazed, and that is undoubtedly one of the most incredible classes I have had, and that definitely influenced me…” (S116, male, 18 years old, CG)*
	Digital media	*“I have always been interested in the area and enjoyed studying and knowing curiosities of the human body, even more with the popularization of educational videos on YouTube”. (S22, male, 20 years old, IG)*
	Health Policies	*“Because of my admiration for primary care and my taste for science, I decided to choose a medical career”. (S90, male, 20 years old, CG)*
	Volunteering	*“I had the opportunity to follow a day of consultations with a group of homeless. It was an incredible experience that made me reflect on how medical practice goes beyond health environments”. (S127, female, 20 years old, CG)*

The second category, “Role models”, included inspiring encounters with teachers/professors, managers, and professionals, as well as the inspiring potential of films and books with medical protagonists in the construction of the students’ imaginaries about the future profession.

The third category, “Previous correlated experiences”, shows the influence of diverse events and contexts, as determinants in the choice of the medical profession. Notably, the students appreciated the possibilities they had to approach medical practice and subjects of this scenario, in guided tours or volunteering, as well as the importance of the health system and universities in bringing the population closer to medical professionals, as shown in the following quote:

*“Because of my admiration for primary care and my taste for science, I decided to choose a medical career*”. (S90, male, 20 years old, CG)

Through analysis of the answers to the question “What are, in your opinion, the main medical profession values?“, seven values and four competences that were relatively more cited could be identified, which showed that the participants had overlapped the two concepts. It was also possible to verify that the intervention group, which had attended a class on the general competences of doctors, referred more frequently to the competences and cited a more significant number of values than the control group, which had attended a biochemistry class. The most cited values were empathy, humility, and ethics; the main competences were technical competence, communication/active listening, and resilience (Table 2).

The answers to the question “How do you imagine yourself working as a medical doctor ten years after graduation?” informed the generation of three categories: “Academic activity”, “Assistance activity”, and “Personal-Professional Life Balance” (Table 3). In the category “Academic activity”, it was observed that the newcomers perceived and valued academic activity as a way to positively impact society. The idea that knowledge should be shared was also frequently mentioned, as shown by this participant:

*“I will seek to become a university professor and be able to positively impact the education of younger people”.* (S94, male, 33 2*years old*, CG)

**Table 2:** Analysis of responses to the question “What are, in your opinion, the main medical profession values?”

Values	Number of students	Intervention Group N (%)	Control Group N (%)	P value
Empathy	46	32 (69.5%)	14 (30.4%)	0.005
Humility	30	20 (66.6%)	10 (33.3%)	NS
Ethics	28	22 (78.5%)	6 (21.4%)	0.003
Humanism	16	12 (75.0%)	4 (25.0%)	NS
Respect	23	17 (73.9%)	6 (26.0%)	0.031
Solidarity	19	13 (68.4%)	6 (31.5%)	NS
Social Responsibility	9	5 (55.5%)	4 (44.4%)	NS
**Competences**				
Technical competence	45	24 (53.3%)	21 (46.6%)	NS
Communication/ Active listening	35	28 (80.0%)	7 (20.0%)	< 0.001
Resilience	13	7 (53.8%)	6 (46.1%)	NS
Collaboration/ Leadership	9	8 (88.8%)	1 (11.1%)	0.045

The category “Assistance activity” shows that the entrant to medical school has a multifaceted vision of his future, which includes both activities to improve the public health system and highly specialized well-paid work. The items “Recognition” and “Success” complement each other and reinforce the idea of measuring success in terms of serving those in need and being well paid. The item “Professionalism” brings the desire to practice medicine based on the values and skills mentioned above, as shown in the following quote:

*“I envision myself providing ethical, benevolent, and empathetic care to all types of patients in need of a doctor”.* (S30, male, 18 *years old*, IG)

The third category, “Personal-Professional Life Balance”, includes statements about self-care practices, the development of hobbies, and reduced working time. However, few students’ responses were classified in this category (11 students).

**Table 3:** Qualitative analysis of responses to the question “How do you imagine yourself working as a medical doctor ten years after graduation?”

Categories	Items	Sample Speeches
Academic activity	Research and Teaching	*“I will seek to become a university teacher and be able to positively impact the education of younger people”. (S94, male, 33 years old, CG)*
Assistance activity	Professionalism	*“I envision myself providing ethical, benevolent, and empathetic care to all types of patients in need of a doctor”. (S30, male, 18 years old, IG)*
	Recognition	*“I hope I have built a reputation so that even before someone comes in to consult with me, they already know they can put their trust in me”. (S133, male, 24 years old, CG)*
	Success	*“A successful professional, both financially and professionally, and above all, I hope to find my role in society in a way that I have positively influenced the community”. (S5, male, 18 years old, IG)*
Personal-Professional Life Balance	Quality of life	*“In a comfortable position in my career, sleeping eight hours a night…” (S128, female, 17 years old, CG)*

The analysis of the answers to the question “How did the COVID-19 pandemic influence your view of the medical profession and your future practice?” informed the generation of three categories: “Professional training”, “Resignification of the profession”,, and “Purpose” (Table 4).

**Table 4:** Qualitative analysis of responses to the question “How did the COVID-19 pandemic influence your view of the medical profession and your future practice?”

Categories	Items	Sample Speeches
Professional training	Evidence-based practice	“*It boosted in a very intense way my desire to always want to be updated, to practice medicine based on scientific evidence”.* (S41, male, 19 *years old*, IG).
	Soft skills	*“The doctor alone cannot promote the health process in its magnitude, so it takes a whole team to talk and work together”* (S34, female, 30 *years old*, IG)
Resignification of the profession	Values	“*The pandemic has highlighted the altruistic character and strong moral duty that a doctor should have.*” (S22, male., 22 *years old*, IG)
		“*It made me value health professionals even more, mainly because there are examples in my home. Seeing their courage, commitment, and dedication during this period encouraged me even more to aim to be a good professional in this area.”* (S9, male, 19 *years old*, IG)
	Vulnerability	*“It has influenced my view of doctors by destroying the superhero doctor image I once held: it has become clear that the most that can be done are what is humanly possible, which often may not be enough”*. (S128, female, 17 *years old*, CG)
	Humanized care	“*The pandemic has reinforced my view of the need for more humane and dignified patient care…”* (S27, male, 26 *years old*, IG)
	Health system	*“The importance of an integrated, complete and public health system […] this challenge has made me an inveterate and unyielding supporter of the SUS.”* (S94, male, 33 *years old*, CG)
Purpose	Meaning of life	“*I realized the role of health professionals goes beyond the profession; it is a purpose you have for other people”.* (S111, female, 18 *years old*, CG)

In the “Professional training” category, it was possible to verify that facing the challenge of a pandemic made students realize the need for constant updating and basing medical practice on scientific evidence. This category also includes statements focusing on skills/attitudes such as resilience, flexibility, and collaboration for teamwork, as illustrated by one participant’s statement:

*“It reinforced my desire to work in medicine, highlighting the importance of the health professional.”* (S4, female, 18 *years old*, IG).

In the category “Resignification of the profession”, the item “Values” highlights the students’ narratives in the previous questions, emphasizing courage and altruism as values demonstrated by health professionals during the pandemic. There were surprising statements about the reconstruction of the “superhero” professional model, which was replaced by a more humane and realistic view of the general limits of medical practice and medicine. One student made the following remark:

“*It has influenced my view of doctors by destroying the superhero doctor image I once had; it has become clear that the most that can be done are what is humanly possible, which often may not be enough”.* (S128, female, 17 *years old*, CG).

Additionally, in this category, the item “Health system” brings the recognition that medical practice is done in the system and for the health system, which in Brazil is represented by the public and universal Unified Health System (SUS), which did organize and guarantee access to free care during the pandemic.

The category “Purpose” included statements that highlighted the pandemic events as the main catalysts for students to understand the role of medicine in their lives and the meaning that care would give them.

“*I realized the role of health professionals goes beyond the profession; it is a purpose you have for other people”.* (S111, female, 18 *years old*, CG)

An analysis of the levels of reflection, which included diffuse description, objective description, emotional exploration, and reflection with commitment, was carried out in both groups. For comparison between the two groups of students, we clustered the categories of lower levels of reflection (diffuse description and objective description) and the categories corresponding to higher levels of reflection (emotional exploration and reflection with commitment) (Table 5). There was a predominance of reflections with a higher level in the intervention group. In contrast, in the control group, descriptions with a lower level of reflection predominated in the three questions analyzed.

**Table 5:** Level of self-reflection in the two groups of medical students

	Control Group (*n* = 78)		Intervention Group (*n* = 81)		P value
	Scant and avoidant reporting + objective reporting	Emotional exploration + committed reflection	Scant and avoidant reporting + objective reporting	Emotional exploration + committed reflection	
Question 1	47	30	33	45	0.030
Question 3	58	18	37	43	< 0.001
Question 4	41	37	27	54	0.022

Question 1: “Tell us something that happened to you in your life that influenced your view of the medical profession.”

Question 3: “How do you imagine yourself working as a medical doctor ten years after graduation?”

Question 4: “How did the COVID-19 pandemic influence your view of the medical profession and your future practice?”

## Discussion

Several authors in the field of adult education, such as Malcolm Knowles, Lev Vygotsky, and Paulo Freire, consider it fundamental for meaningful and effective learning to know what the learner already knows or has experienced [20–22]. This view of education was the framework for our study. Considering the formation of an ethical professional identity, with the values of the profession being fundamental for medical training and society, we studied the vision of the medical profession that medical students bring when they enter medical school.

We invited students who had just entered medical school to describe their views on questions related to critical incidents, values of the medical profession, expectations of the future professional, and influences of the COVID-19 pandemic on their choice to practice medicine. The questions were designed neutrally, without the intention to induce either positive or negative responses. It should be noted that the timing of data collection, still during the COVID-19 pandemic, must have influenced the observed results. We analyzed how the experiences lived until their entry into university transformed their vision of the medical professional they would like to build, as well as the impact observed in an isolated pedagogical intervention in proposing reflection and changes in developing their professional identity.

According to Charon, we can understand the meaning of narratives through cognitive, symbolic, and affective means [23]. When the students were encouraged to narrate a significant event (critical incident) that motivated them to choose the medical profession (question one), a particular emphasis was given to the category “Process of illness and care”, with recollections of illness experiences in their personal lives and family spheres that influenced their choice of the medical profession. Notably, illness experiences were presented as critical or memorable incidents, which are defined by Mezirow [24] as transformative events capable of promoting critical and profound reflections, leading to paradigm shifts in the construction of professional identity.

According to Flanagan [25], an incident is any observable human activity that is sufficiently complete, in itself, to allow inferences about the person performing the action. To be critical, an incident must occur in a situation in which its influence is apparent to the observer, leaving no doubt about its effects.

Branch [12] describes that critical incident reports are ideal for assessing values and attitudes and teaching professional development, endorsing the tool as effective for describing one’s professional experiences.

Additionally, on question one, it is worth mentioning that the professional models that existed before entering medical school strongly influence the design of the construction of being a doctor, as they act as guides of professional transformation, as proposed by Gardeshi et al. [26] and Brennan et al. [1]. According to Cruess et al. [27], role models are mirrors of technical values and ethical and humanistic principles for a future doctor, allowing him or her to create a critical basis necessary for decision-making in his or her professional life.

Regarding the fundamental values of the medical profession (question two), we observed that students already bring values with them, even those who recently started school, as Reimer et al. [28] proposed. However, the intervention group referred more to values and competences than the control group, denoting that the intervention had a positive impact in stimulating a deeper reflection about their professional identity. Students in the intervention group cited empathy, humility, ethics, humanism, respect, and solidarity as medical professional values more than students in the control group.

A pedagogical intervention is an interference in the teaching-learning process that is applied by an educator with the goal of overcoming obstacles and optimizing the construction of knowledge, in addition to producing impact and promoting changes in directions for the future [29]. In the present study, we analyzed and compared the impact of students’ exposure to medical professionalism content in the intervention group to a control group. For Holden et al. [7], multiple interventions during their training, even if punctual, will contribute to the dynamic process of professional identity formation.

When we asked the students how they imagine practicing medicine ten years after graduation (question three), some could not envision the future scenario, perhaps because they were just starting medical school. However, many cited academic life as a way to impact the training of new young people. Another possible path expressed in the category “assistance” was based on humanistic professional values, simultaneous involvement with the private and public health system, and the construction of professional and financial recognition. Quality of life and balance between professional and personal life were addressed as a theme in only a few narratives.

The COVID-19 pandemic had a significant impact on Brazil, which was the second country in the world in terms of the number of deaths [30]. All the students who answered the questions spent approximately two years living with health restrictions and taking classes almost exclusively remotely [31]. It is essential to assess the impact of this whole experience on the vision of the medical professional identity of these students.

To evaluate the answers to question four, “How did the COVID-19 pandemic influence your view of the medical profession and your future practice?”, we must consider that the occurrence of a significant event, such as the COVID-19 pandemic, strongly influenced the statements about finding a true purpose for future professional life and the discovery of a medicine based on ethical values. It should be highlighted in the quotes how much the pandemic catalyzed the need to reflect on scientific and technical qualifications, teamwork, and multiple fronts. From the students’ perspective, the impact of the pandemic was positive in terms of the values of the profession as a service to society and teamwork and even in terms of the decision to pursue a career as a doctor.

How medical students reflect on the critical incidents that occur during their academic training has great value in the formation of their medical identity. The greater their ability to reflect deeply and critically on events is, the more transformative their learning will be, which is why many educational institutions have incorporated training in their curricula to increase the reflective practice of future professionals [32–35].

In addition to the analysis of the categories, the answers to questions one, three, and four were also analyzed regarding the level of reflection developed by each student. The scale of reflective complexity proposed by Niemi [19] was used as a tool for analyzing reflections.

In the analysis of the totality of the reflections of the three questions, we noticed a predominance of reflections of the objective description (OD) and diffuse description (DD) type, that is, descriptions with less reflection and critical capacity among the students who did not attend the conference on professionalism and who constitute the group that did not have any contact with the discussion of the values of the profession within the medical course. This fact is in line with the results of Montgomery [36], who observed a design with a preponderance of low levels of reflection in students in the preclinical phase, as only 45.7% of the students were able to issue reflective reports when describing the proposed questions. For Niemi [19], the scarcity of critical reflections found among groups of students in the preclinical phase of the medical course is justified by the absence of memorable critical experiences or incidents, which prevents them from envisioning their “professional self” at a future point in time [37]..

When we evaluated the three questions in terms of differences between the intervention and control groups, we observed that reflections of the objective description (OD) and diffuse description (DD) type predominated in the control group; the opposite occurred in the intervention group, in which there was a predominance of more elaborate, reflective, and committed descriptions (RC) or emotional exploration (EE). This finding reflects the influence of the sensitization content on the intervention group, suggesting that the process of reflecting is a skill to be developed [13].

Each medical student newcomer to school brings with him or her a preconceived notion of expectations about the medical profession. However, his or her ability to reflect on professionalism changes according to the phase of his or her training cycle [28]. In the present study, we were able to measure that an isolated and well-structured pedagogical intervention was a tool that was capable of promoting an impact on the students’ view of professionalism. Nevertheless, we cannot assess the intensity and duration of this effect since multiple interventions are necessary to build the professional identity of future doctors.

In a systematic review on strategies for developing medical professionalism among future doctors, Passi et al. [8] noted that there are no clear guidelines on the most effective strategies to help medical students develop high levels of medical professionalism. The studies they selected identified five critical areas for this process: curriculum design, student selection, teaching/learning methods, mentoring (role modeling), and assessment methods. There is evidence that mentors and the hidden curriculum are more important for professional identity formation than formal activities such as conferences [4]. However, in our study, we demonstrated the impact of a single, formal activity on students’ values of the profession and capacity for self-reflection.

## Conclusion

In conclusion, our study showed that medical students, upon entering medical school, already have an adequate view of medical professionalism, although they still need to present a deeper level of self-reflection. A program intervention in medical professionalism can promote self-reflection. The vision of medical professional identity was strongly influenced by the COVID-19 pandemic, positively impacting the formation of a professional identity among the students who decided to enter medical school.

## Data Availability

The datasets generated during and/or analyzed during the current study are available from the corresponding author on reasonable request.

## References

[1] Cohen JJ (2006). Professionalism in medical education, an American perspective: from evidence to accountability. Med Educ.

[2] Cruess RL, Cruess SR, Johnson SE (2000). Professionalism and medicine’s social contract. J Bone Joint Surg Am.

[3] Brennan T, Blank L, Cohen J, Kimball H, Smeler N, Copeland R (2002). Project of the ABIM Foundation, ACP–ASIM Foundation, and European Federation of Internal Medicine. Medical professionalism in the new millennium: a physician charter. Ann Intern Med.

[4] Lehmann LS, Sulmasy LS, Desai S, ACP Ethics, Professionalism and Human Rights Committee (2018). Hidden curricula, ethics, and professionalism: optimizing clinical learning environments in becoming and being a physician: a position paper of the American College of Physicians. Ann Intern Med.

[5] Cruess SR, Cruess RL (2008). Understanding medical professionalism: a plea for an inclusive and integrated approach. Med Educ.

[6] Cruess RL, Cruess SR, Steinert Y (2016). Amending Miller’s pyramid to include professional identity formation. Acad Med.

[7] Holden MD, Buck E, Luk J, Ambriz F, Boisaubin E, Clark M (2015). Professional identity formation: creating a longitudinal framework through TIME (transformation in medical education). Acad Med.

[8] Passi V, Doug M, Peile E, Thistlethwaite J, Johnson N (2010). Developing medical professionalism in future doctors: a systematic review. Int J Med Educ.

[9] Siqueira MAM, Torsani MB, Gameiro GR, Chinelatto LA, Mikahil BC, Tempski PZ (2022). Medical students’ participation in the Volunteering Program during the COVID-19 pandemic: a qualitative study about motivation and the development of new competencies. BMC Med Educ.

[10] Tempski P, Arantes-Costa FM, Kobayasi R, Siqueira MAM, Torsani MB, Amaro BQRC et al. Medical students’ perceptions and motivations during the COVID-19 pandemic. PLoS ONE. 2021: e0248627. 10.1371/journal.pone.0248627 PMC7968644 33730091

[11] Frank JR, Snell L, Sherbino J, Boucher A. CanMEDS 2015. Physician competency framework series I. 2015.

[12] Branch WT (2005). Use of critical incident reports in medical education, a perspective. J Gen Inter Med.

[13] Sandars J (2009). The use of reflection in medical education: AMEE Guide 44. Med Teach.

[14] Branch WT, Pels RJ, Lawrence RS, Arky RA (1993). Becoming a doctor: ‘‘critical-incident’’ reports from third-year medical students. N Engl J Med.

[15] Tong A, Sainsbury P, Craig J (2007). Consolidated criteria for reporting qualitative research (COREQ): a 32-item checklist for interviews and focus groups. Int J Qual Health Care.

[16] Denzin NK (1978). The research act: a theoretical introduction to sociological methods.

[17] Denzin N, Lincoln YS (2003). The landscape of qualitative research: theories and issues.

[18] Patton MQ (1990). Qualitative evaluation and research methods. 2ed.

[19] Niemi PM (1997). Medical student’ professional identity: self-reflection during the preclinical years. Med Educ.

[20] Freire P (2012). Pedagogy of hope: reliving pedagogy of the oppressed.

[21] Knowles MS, Holton EF III, Swanson RA, Swanson S, Robinson PA. The adult learner: The definitive classic in adult education and human resource development. Taylor & Francis. London. 9th Edition. 2020.

[22] Newman S, Vygotsky (2018). Wittgenstein, and sociocultural theory. J Theory Soc Behav.

[23] Charon R (2001). Narrative Medicine: a model for empathy, reflection, profession and trust. JAMA.

[24] Mezirow J. Transformative dimensions of adult learning. San Francisco. Jossey-Bass,; 1991.

[25] Flanagan JC (1954). The critical incident technique. Psychol Bull.

[26] Gardeshi Z, Amini M, Nabeiei P (2018). The perception of hidden curriculum among undergraduate medical students: a qualitative study. BMC Res Notes.

[27] Cruess SR, Cruess RL, Steinert Y (2008). Role modelling - making the most of a powerful teaching strategy. BMJ.

[28] Reimer D, Russell R, Khallouq BB, Kauffman C, Hernandez C, Cendán J (2019). Pre-clerkship medical students’ perceptions of medical professionalism. BMC Med Educ.

[29] Cianciolo AT, Regehr G (2019). Learning theory and educational intervention: producing meaningful evidence of impact through layered analysis. Acad Med.

[30] World Health Organization Coronavirus (COVID-19) Dashboard. 2023. https://covid19.who.int/. Acessed June 27, 2023.

[31] Chinelatto LA, Costa TR, Medeiros VMB, Boog GHP, Hojaij FC, Tempski PZ (2020). What you gain and what you lose in COVID-19: perception of medical students on their education. Clinics.

[32] Andersen RS, Hansen RP, Søndergaard J, Bro F (2008). Learning based on patient case reviews: an interview study. BMC Med Educ.

[33] Boenink AD, Oderwald AK, De Jonge P, Van Tilburg W, Smal JA (2004). Assessing student reflection in medical practice. The development of an observer-rated instrument: reliability, validity and initial experiences. Med Educ.

[34] Man K, Gordon J, MacLeod A. (2009). Reflection and reflective practice in health professions education: A systematic review. Adv Health Sci Educ. 2009; 14:595–621. 10.1007/s10459-007-9090-2 18034364

[35] Plack MM, Driscoll M, Marquez M, Greenberg L (2010). Peer-facilitated virtual action learning: reflecting on critical incidents during a pediatric clerkship. Acad Pediatr.

[36] Montgomery A, Doulougeri K, Panagopoulou E (2021). Do critical incidents lead to critical reflection among medical students?. Health Psychol Behav.

[37] Brady DW, Corbie-Smith G, Branch J, William T (2002). What’s important to you? The use of narratives to promote self-reflection and to understand the experiences of medical residents. Ann Inter Med.

